# *Lactobacillus rhamnosus* GG as dietary supplement improved survival from lipopolysaccharides‐induced sepsis in mice

**DOI:** 10.1002/fsn3.2630

**Published:** 2021-10-15

**Authors:** Ko‐Chung Tsui, Ting‐Lin Yen, Chi‐Jung Huang, Kun‐Jing Hong

**Affiliations:** ^1^ Department of Medical Research Cathay General Hospital Taipei Taiwan; ^2^ Division of Infectious Diseases Department of Internal Medicine Cathay General Hospital Taipei Taiwan; ^3^ Department of Clinical Pathology Cathay General Hospital Taipei Taiwan; ^4^ School of Medicine Fu Jen Catholic University New Taipei City Taiwan; ^5^ Department of Oral Hygiene Care Ching Kuo Institute of Management and Health Keelung Taiwan

**Keywords:** Firmicutes/Bacteroidetes ratio, gut microbiota, *Lactobacillus rhamnosus* GG, lipopolysaccharides, sepsis

## Abstract

Sepsis is a state of host immune response triggered by virus or bacterial infection, in which the extent of regional and systemic inflammation and companion counter‐inflammatory reactions determines disease outcomes. Probiotics are known for the immunomodulatory effect on allergic disorders, but it is not clear whether the beneficiary effect extends to sepsis and increases survival. In this mouse model, we injected intraperitoneally lipopolysaccharides (LPS) to induce sepsis, and investigated whether the pretreatment of *Lactobacillus rhamnosus* GG (LGG) contributed to host survival and examined the alteration of the gut microbiota and blood cytokines/chemokines profile before sepsis induction. Four‐week‐old male BALB/c mice were divided into two groups: one group were fed daily with LGG as a dietary supplement for fourteen days, whereas the other group with sterile water. Before sepsis induction, some mice from each group were killed to collect stool in the intestine and blood for microbial metagenomic and cytokine/chemokine analyses, respectively, and the rest were monitored afterward for mortality. The relative abundance of several families in the gut microbiota after LGG treatment was altered as well as the ratio of Firmicutes/Bacteroidetes. In addition, several pro‐inflammatory cytokines such as G‐CSF, IL7, IL15, and MCP1 were lower in the LGG group than in the control group. The survival rate following LPS‐induced sepsis improved with LGG treatment. Our results indicated that dietary supplement of probiotic LGG improved survival from LPS‐induced sepsis, most likely through pre‐septic changes in the gut microbial constituents by LGG with reciprocal alteration of host immune system to a less reactive state to incoming pathogens.

## Graphical abstract

LGG as dietary supplement decreased the F/B ratio of the gut microbiota and down‐regulated several pro‐inflammatory cyto‐kines/chemokines, but also increased survival rate in response to experimental sepsis.

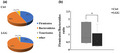

## 1 INTRODUCTION

Sepsis is a state of host immune response to bacteria or virus infection, manifesting in various signs and symptoms as a result of locoregional as well as generalized inflammatory reactions (Cohen, 2002). Recent studies indicated that the prognosis of sepsis was not entirely determined by toxins of pathogens. In fact, host inflammatory responses to pathogens may play even more important roles during sepsis. In the early phase of sepsis, host response to pathogens results in a state of hyperinflammation characterized by excessive proinflammatory cytokines, including interleukin 1 (IL‐1) and tumor necrosis factor‐alpha (TNF‐alpha) (Bosmann & Ward, 2013; Hotchkiss et al., 2013). If unregulated, the hyperinflammatory state may cause severe tissue damage and place hosts at risk for multiple organ failure and, ultimately, death (Chong & Sriskandan, 2011; Leentjens et al., 2013; Cauwels et al., 2014).

Probiotics are live microorganisms used as a dietary supplement to improve health by maintaining homeostasis in the gut microbial ecosystem (Sánchez et al., 2017). Failure to maintain homeostasis of the ecosystem, either caused by antibiotics or gastrointestinal diseases, is detrimental to general health (Kho and Lal, 2018). In some gastrointestinal diseases such as clostridial colitis or inflammatory bowel diseases, probiotics can accelerate recovery by restoring disturbed gut microbial community back to normal state. It is a common belief that complex interactions between exogenous probiotics and inhabitant microbiota eventually lead to a new balanced state of improved nutritional adaptability.

*Lactobacillus rhamnosus* GG (LGG) is Gram‐positive bacteria known for the ability to survive through harsh gastric acidic environment and adamantly attach to the gut mucosa (Jacobsen et al., 1999; Kankainen et al., 2009; Segers & Lebeer, 2014). As probiotics, LGG effectively alleviated bowel syndrome and reduced intestinal mucosal inflammation in inflammatory bowel disease (Ashraf & Shah, 2014; Han et al., 2019). The anti‐inflammatory activity also extended beyond the gut to reduce symptoms in patients with allergic diseases (Berni‐Canani et al., 2016; Spacova et al., 2019) and atopic dermatitis (Huang et al., 2017). Previous studies showed LGG pre‐treatment reduced intestinal mucosal injury, gut microbita dysbiosis, and sepsis mortality in a mouse model of cecal ligation and puncture (CLP) peritonitis (Chen et al., 2019, 2020). Distant organs such as lung also demonstrated attenuated injury and inflammatory response (Khailova et al., 2014). It was not clear, however, to what extent the alteration of gut microbiota and host immune status before sepsis induction contributed to subsequent tolerance of sepsis, as most studies captured events at least one day into sepsis and thus ongoing reciprocal interactions between the host immune system and the gut microbial community might shift the picture toward a new balanced state representative of interim, not starting, point. In this study, we adopted an LPS‐induced sepsis model, in which host responses would reflect closely to the pre‐septic immune status, and characterized associated changes in the host gut bacterial constituents and blood cytokine/chemokines by LGG treatment before septic indution.

## 2 MATERIALS AND METHODS

### 2.1 LPS‐induced sepsis model and survival analysis

Four‐week‐old male BALB/c mice were purchased from BioLasco (Taipei, Taiwan). Mice with an average weight of 18 g were housed for at least a week prior to use. For survival study, mice were randomly divided into two groups according to whether LGG was added as a dietary supplement: control group (*n* = 14) received 200 µl sterilized water by oral gavage and LGG group (*n* = 14) received 200 µl suspension of *Lactobacillus casei rhamnosus* GG (8*10^7^ CFU; Anti‐biophilus Capsules, Laboratoires Lyocentre). Mice were fed for 14 days before sepsis induction by intraperitoneal injection of 20 mg/kg of LPS from *Escherichia coli* O55:B5 (Sigma‐Aldrich‐L2880) and then monitored at 6th, 12th, 24th, 48th, and 72th h afterward for survival or death. The animal use protocol was reviewed and approved by the Cathay General Hospital of the Institutional Animal Care and Use Committee (IACUC), protocol number 109–019, approval number IACUC109‐019.

### 2.2 Fecal DNA extraction

To prepare fecal samples for microbiota analysis, mice were fed the same way as those in the survival study and killed to retrieve feces from the small intestine after fourteen days. Each sample prepared for DNA extraction was composed of feces recovered from two to three mice in one experiment to ensure adequate quantity, and the final microbial diversity results representative of mice in the LGG or Ctrl group were based on the average of diversity data obtained from five repeated experiments, or twelve mice in total, for either group. Genomic DNA of fecal microbiome was extracted with QIAamp Fast DNA Stool Mini Kit (Qiagen). All procedures were performed according to the manufacturer's instructions. Stool samples were lysed with inhibitEX buffer and homogenized in FastPrep‐24 5G (MP biomedicals) until dissolved. The supernatants were digested with proteinase K and then processed with the QIAamp mini spin columns. The retained DNA on silica membranes was recovered with a preheated elution buffer and quantitated by measuring the absorbance at 260 nm with a NanoDrop 2000 spectrophotometer.

### 2.3 16S rRNA sequencing

The sequencing library was constructed by PCR‐amplification of V3–V4 regions in the 16S rRNA gene with KAPA HiFi hotstart readymix (Roche), as described in the protocol of Illumina 16S metagenomics sequencing library preparation. The products were purified by AMPure XP magnetic beads (Beckman Coulter) before labeling with the Nextera XT index kit. The quality of amplified product was evaluated by a Fragment Analyzer. Next, 20% of PhiX control was spiked in 10 pM final pool and the library was sequenced by MiSeq (Illumina). The sequencing was performed on llumina MiSeq instrument with pairedend mode 2 × 300 nt (overlapping reads) using a MiSeq Reagent Kit V3 (600 cycles PE). The cluster density was approximately 800 K/mm^2^. The most passing filter has at least 80% of bases above a Phred score of 30 (Q30), with over 50K reads per sample. Sequencing data were retrieved and analyzed in FASTQ format.

### 2.4 Cytokine/Chemokine assay

Blood samples were collected by cardiac puncture after surgical anesthesia of the mice randomly selected from those killed in the microbiota analysis experiments (*n* = 6 for LGG or Ctrl group) and allowed to completely clot for 30 min at room temperature. The serum was isolated and stored at −20℃ for cytokine/chemokine assay. The concentrations of G‐CSF, GM‐CSF, IFN‐γ, IL1α, IL1β, IL2, IL4, IL5, IL6, IL7, IL9, IL10, IL12 (p40), IL12 (p70), IL13, IL15, IL17, IP10, KC, MCP1, MIP1α, MIP1β, MIP2, RANTES, and TNF‐α were detected by Luminex xMAP^®^ platform following the manufacturer's protocol. Each sample was measured twice and the averages of the repeated measurements were recorded as the result.

### 2.5 Statistical analysis

Numerical data were expressed as mean ± standard deviation. The differences between groups were tested for significance using Student's *t*‐test in SPSS software. A *p* < .05 was considered statistically significant.

## 3 RESULTS

### 3.1 LGG improved body weight and survival rate of mice in LPS‐induced sepsis

Previous studies have shown that LGG reduced fat mass (Ji et al., 2012) and increased survival from CLP peritonitis in C57BL6 mice (Chen et al., 2016). In order to clarify whether different inbred strain or sepsis model could be confounding variables, four‐week‐old male BALB/c mice were fed with LGG (8*10^7^ CFU/200 ml from Antibiophilus capsules) or sterilized water by oral gavage once a day.

**FIGURE 1:**
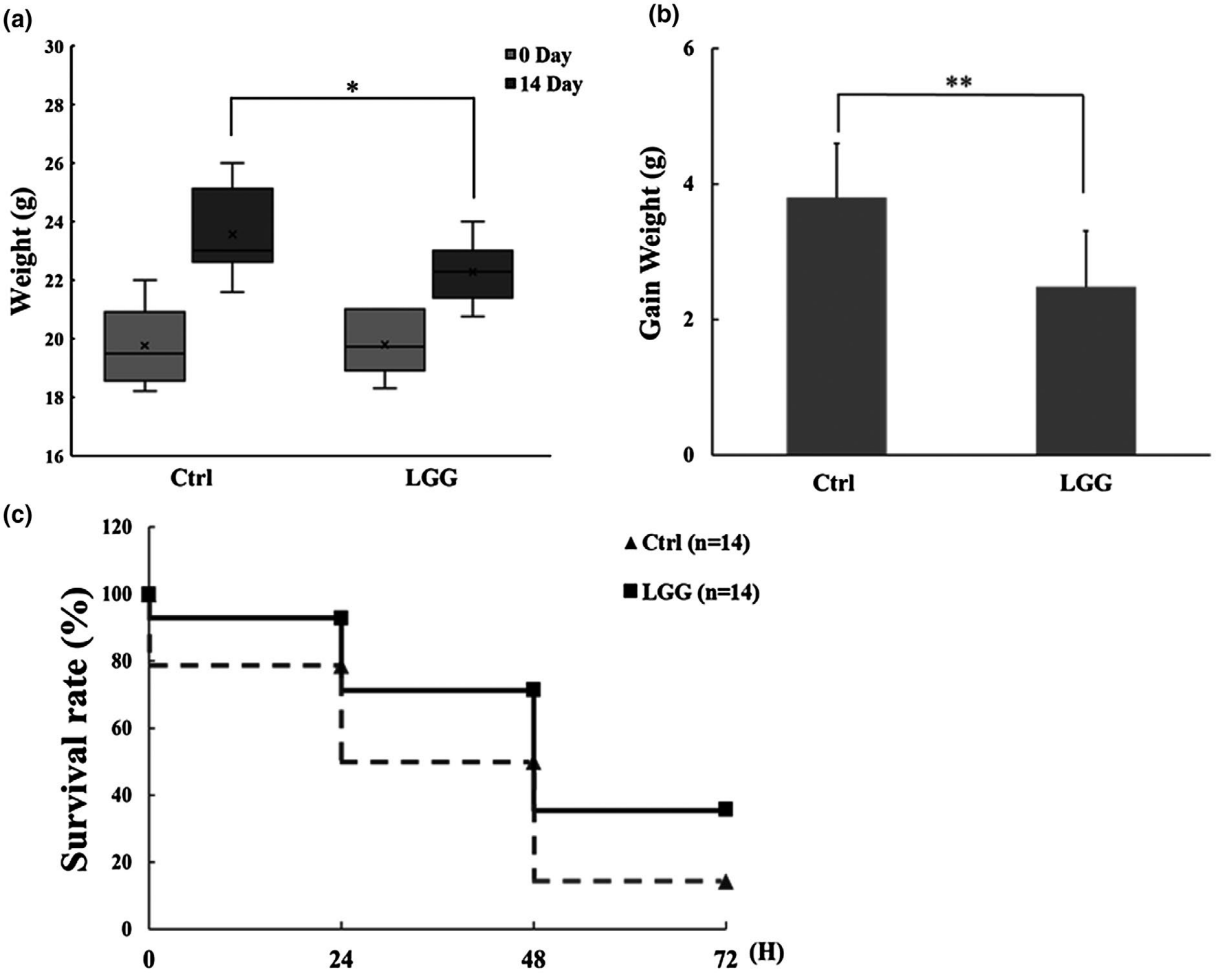
Changes of body weight and survival rate in LPS‐induced sepsis

After 14 days' treatment, mice in the LGG group had lower body weight gain than those in the Ctrl group (Figure 1a), with means of 2.48 ± 0.82 g for the mice in the LGG group and 3.8 ± 0.8 g for those in the control group (*n* = 14 for either goup, *p* <.01, Figure 1b). Next, we determined whether taking LGG once daily has a better survival rate from LPS‐induced sepsis. The mice were given 20 mg/kg of LPS by intraperitoneal injection and followed up at 6th, 12th, 24th, 48th, and 72th hours. As shown in Figure 1c, there was a significant improvement in survival between mice in the LGG and those in the Ctrl group. Similar results were also observed in ICR mice (Figure S1). Therefore, these data indicated that LGG once daily provided protection against LPS‐induced sepsis.

### 3.2 LGG affected on the gut microbiota and the Firmicutes/Bacteroidetes ratio

**FIGURE 2:**
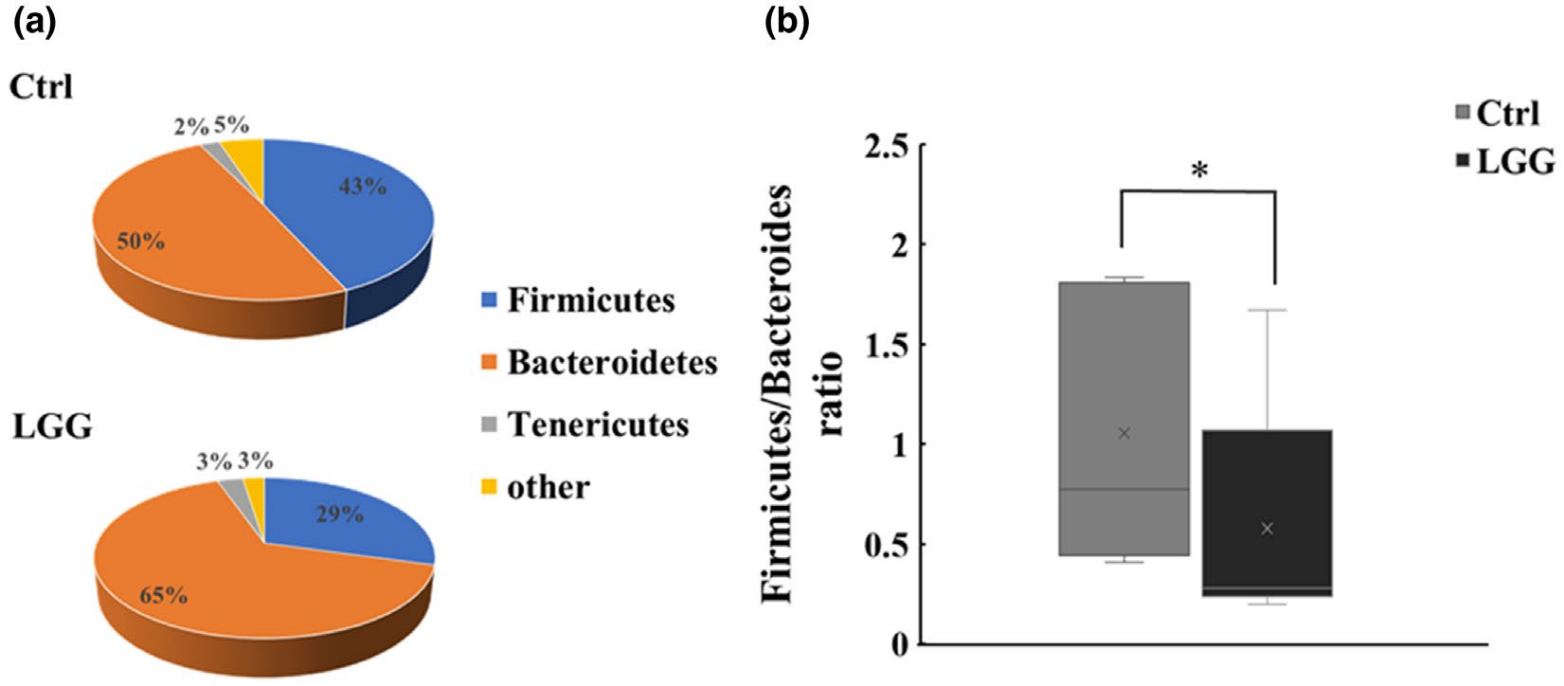
The effect of LGG on the gut microbiota at phylum level

In order to study the effect of LGG on the microbial community in the gut, we collected residual stool from mice intestine with or without treatment of LGG. The total of 1,752,971 sequencing reads for gut microbiota were detected with an average of the numbers of 187,324 (Ctrl group, *n* = 5) and 163,269 (LGG group, *n* = 5) read, respectively. Each sample contained residual stool from two to three mice. The major phyla of intestinal microbiota were shown in Figure 2a. The relative abundances of Firmicutes (43% in Ctrl versus 29% in LGG) and Bacteroidetes (50% in Ctrl versus 65% in LGG) changed with LGG treatment, but not for Tenericutes (2% in Ctrl versus. 3% in LGG).

**FIGURE 3:**
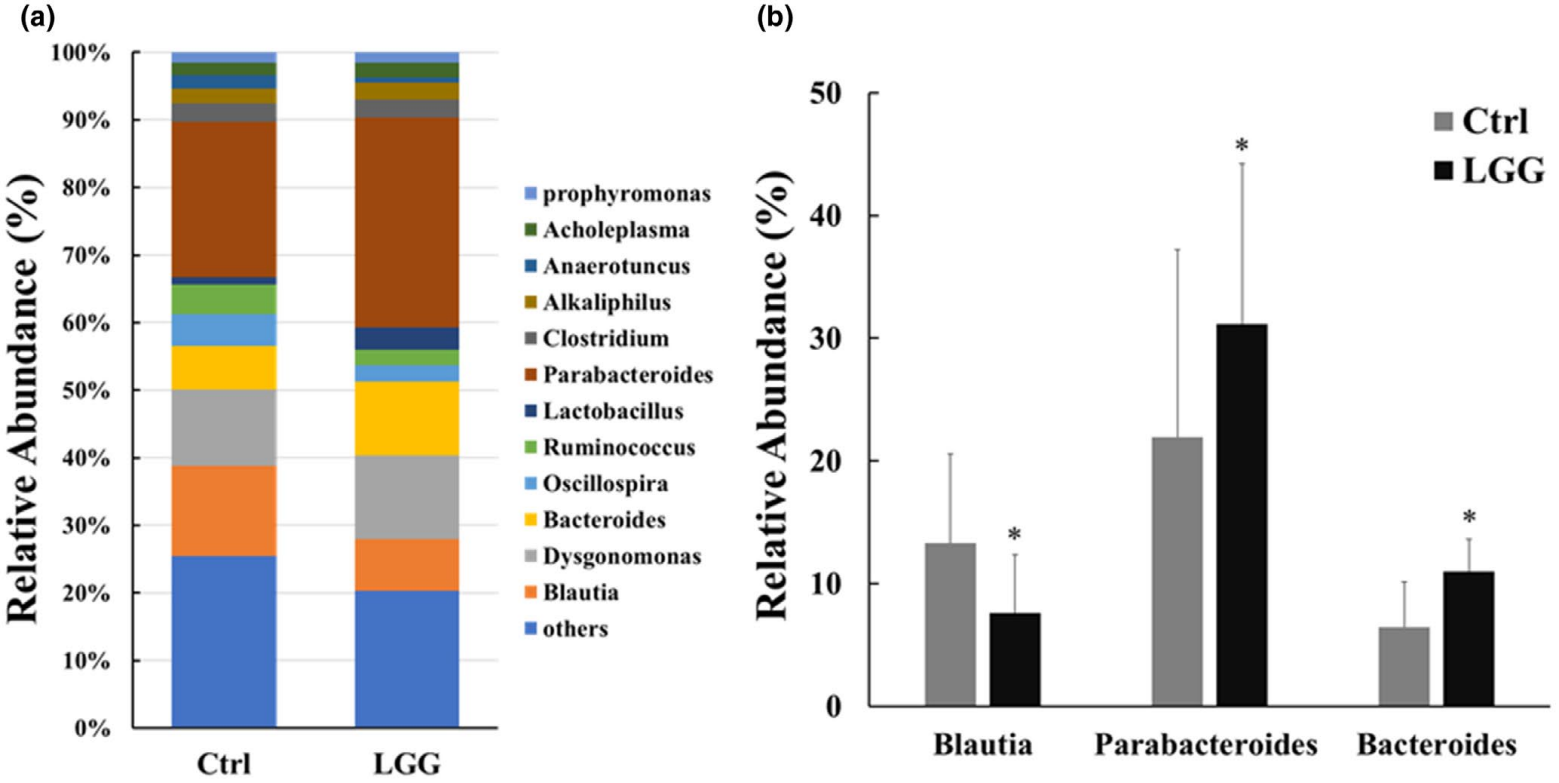
The effect of LGG on the gut microbiota at genus level

Firmicutes and Bacteroidetes were regarded as important regulators of human gut microbiota (Qin et al., 2010). Multiple studies reported that Firmicutes to Bacteroidetes ratio (F/B ratio) correlated with several pathological conditions or diseases (Magne et al., 2020; Mariat et al., 2009; Vaiserman et al., 2020). Therefore, we calculated the ratio of the relative sequence abundances of Firmicutes to Bacteroidetes. As shown in Figure 2b, the F/B ratio was significantly decreased in the LGG group compared with the control group (*p* < .05). Further characterization of stool microbiota at genus level demonstrated unequal impacts by LGG on the abundances of different bacteria (Figure 3a), in which only Blautia, Parabacteroides, and Bcateroides were significantly affected by LGG (*p* < .05, Figure 3b). The relative abundances of Parabacteroides and Bcateroides were up‐regulated by LGG, whereas for Blautia it was down‐regulated. The result implied that Blautia, Parabacteroides, and Bcateroides may assume different roles in adjusting host immune status.

### 3.3 LGG changed the measurement of Cytokine/Chemokine in mice

To evaluate changes in the profile of cytokine/chemokine with LGG treatment, we used a MILLIPLEX MAP Mouse Cytokine/Chemokine Magnetic Bead Panel‐ Immunology Multiplex Assay to quantitate cytokines/chemokines in the serum. The mean concentrations of each chemokine and cytokine were shown in Table 1. Among all 25 cytokines examined, only four cytokines, namely, G‐CSF (348.99 ± 124.42 pg/ml in Ctrl versus 174.49 ± 61.54 pg/ml in LGG, *p* <.05), IL7 (4.16 ± 0.95 pg/ml in Ctrl versus 2.49 ± 0.26 pg/ml in LGG, *p* <.01), IL15 (23.99 ± 5.91 pg/ml in Ctrl versus 6.64 ± 7.70 pg/ml in LGG, *p* <.01), and MCP1 (33.30 ± 27.08 pg/ml in Ctrl versus 4.83 ± 6.29 pg/ml in LGG, *p* <.05), were significantly decreased in the LGG group compared to controls.

**TABLE 1:** Summary of Cytokine/Chemokine analysis data

Cytokine/Chemokine	Ctrl *N* = 6 (pg/ml)	LGG *N* = 6 (pg/ml)	*p*
G‐CSF	348.99 ± 124.42	174.49 ± 61.54	.017*
GM‐CSF	22.77 ± 15.73	14.77 ± 12.03	.35
INF‐r	4.08 ± 0.4	4.18 ± 0.36	.64
IL−1a	254.80 ± 45.85	292.87 ± 134.95	.54
IL−1b	6.29 ± 3.24	3.74 ± 0.73	.11
IL−2	3.79 ± 0.62	3.10 ± 0.67	.09
IL−4	3.09 ± 0.32	2.92 ± 0.2	.3
IL−5	8.48 ± 2.43	7.80 ± 2.33	.63
IL−6	3.96 ± 0.5	3.57 ± 0.97	.4
IL−7	4.16 ± 0.95	2.19 ± 0.26	<.01**
IL−9	86.08 ± 32.95	78.06 ± 20.66	.62
IL−10	3.84 ± 1.14	3.26 ± 1.21	.41
IL−12(p40)	6.14 ± 0.92	7.10 ± 2.6	.42
IL−12(p70)	12.93 ± 7.03	8.66 ± 10.14	.41
IL−13	50.56 ± 9.41	43.66 ± 7.4	.19
IL−15	23.99 ± 5.91	6.64 ± 7.7	<.01**
IL−17	9.02 ± 4.94	8.47 ± 4.9	.84
IP−10	171.86 ± 58.15	230.32 ± 94.44	.23
KC	54.74 ± 33.74	76.68 ± 31.66	.27
MCP−1	33.30 ± 27.08	4.82 ± 6.3	.04*
MIP−1a	31.78 ± 5.47	27.61 ± 12.26	.47
MIP−1b	72.82 ± 17.38	68.67 ± 19.26	.70
MIP−2	113.26 ± 31.97	97.67 ± 21.28	.35
RANTES	13.84 ± 10	18.62 ± 11.44	.46
TNF‐a	7.1 ± 2.74	5.34 ± 0.6	.18

Note

The results were expressed as mean ± standard deviation. Ctrl, control; LGG, *Lactobacillus rhamnosus* GG.

## 4 DISCUSSION

This study demonstrated that LGG as a dietary supplement for fourteen days increased survival from LPS‐induced sepsis in mice, with companion changes in the baseline gut microbiota and cytokine profile. In this study, we analyzed gut microbiome in fecal material obtained directly from the intestine instead through defecation. The advantage of analyzing in situ fecal sample includes minimizing deterioration of the constituents in the gut microbiota and thus bias for microbiome profiling (Zmora et al., 2018). Therefore, we believe that the changes in the relative abundance of different bacteria reliably reflect the impact of probiotic LGG on the gut microbial community.

There were several interesting observations in this study. First, LGG significantly lowered the ratio of Firmicutes to Bacteroidetes (F/B). Firmicutes and Bacteroidetes are two most dominant bacterial phyla in the gut microbiota, representing over 90% of the total bacterial load (Human Microbiome Project Consortium, 2012; Qin et al., 2010). Firmicutes are related with energy harvest and storage, whereas Bacteroidetes have capacity for energy consumption (Turnbaugh et al., 2006). The F/B ratio has been used as an index of nutritional adaptability and obesogenic property of the gut microbiota (Castaner et al., 2018; Gohir et al., 2015; Li & Ma, 2020). Indeed, the mice in the LGG group gained less weight than those in the control group (Figure 1b), suggesting decreased population of Firmicutes in the gut microbiota led to less energy harvest and thus less fuel into the inflammatory furnace during the hyperinflammatory phase of sepsis. Interestingly, Chen et al. found that the dietary supplement of LGG increased the abundance of Firmicutus and F/B ratio one day after cecal ligation and puncture (CLP) procedure (Chen et al., 2019). The observed change in the relative abundance of microbial constituents may derive from reciprocal interactions between the gut micrbiome and ongoing intestinal inflammation, or was simply due to sampling site difference, as F/B ratio was lower in feces recovered from the small intestine than through defecation (Ji et al., 2012). Second, although feeding on LGG increased the relative abundance of Lactobacillus spp. in the gut microbiome, Parabacteroides and Bacteroides were among the most abundant bacteria that significantly increased after LGG treatment (Figure 3b). Of note, Parabacteroides spp. were able to derive metabolic benefits to help reduce weight gain, hyperglycemia, and hepatic steatosis in high‐fat diet (HFD)‐fed mice (Wang et al., 2019). In addition, Parabacteroides spp. attenuated chemically induced gut epithelial inflammation in mice and were thought to assume a role in blocking inflammation (Kverka et al., 2011). Therefore, it is likely that the observed weight reduction and sepsis survival advantage in our study were due to a global change in the microbial community of the gut with improvement in nutrients utilization and inflammatory responses.

As a dietary supplement, LGG has been reported to confer survival advantage in CLP peritonitis models (Chen et al., 2016, 2019; Khailova et al., 2013) with results similar to ours (Figure 1c). Although CLP‐induced sepsis model arguably was more physiologically relevant than by simple LPS injection, the combination of sequential insults from laparotomy trauma, ischemic tissue of the ligated cecum, and peritoneal fecal spillage complicated interpretation of survival data as well as temporal profiles of inflammatory cytokines (Lewis et al., 2016). On the other hand, bolus administration of LPS essentially neglects the host‐pathogen interactions and non‐septic inflammatory responses, and the survival data can be interpreted in the context of pre‐septic host immune status to endotoxin. In fact, the concentration of four cytokines, namely, G‐CSF, IL7, IL15, and MCP1, were significantly lower in LGG‐treated mice than control. In a recent study on cytokine storm, the state of hyperinflammation was associated with high levels of these cytokines (Ruan et al., 2020). The IL7 and IL15 were associated with T‐cell homeostasis and CD4(+) T cell development (Rubinstein et al., 2008). IL15 induced the differentiation of NK cells and proliferation of memory CD8 (+) T cells (Mueller et al., 2005). Compared with wild type, IL15 knockout mice had significantly improved survival in CLP‐induced sepsis, along with reduced NK and CD8+ cells and mitigated sepsis‐induced hypothermia and organ injuries, and the severity of sepsis was restored by exogenous infusion of IL15 owing to activating NK cells (Orinska et al., 2007). MCP1 is an important chemokine for macrophage recruitment and, to the best of our knowledge, we were the first to report MCP1 as one of the down‐regulated cytokines by LGG pretreatment. Several studies demonstrated that MCP1 was an initiating cytokine in inflammatory responses (Deshmane et al., 2009; Wang et al., 2018). Wang et al. (2018) showed that early expression of MCP1 was associated with subsequent development of sepsis in cases of severe trauma and the level of MCP1 could be used as an index of severity. There was also evidence that prebiotics supplement altered the gut microbiota, including an increase in the abundance of Lactobacillus spp., concurrently with reduced expression of MCP1 (Cani et al., 2009). Interestingly, MCP1 deficient mice on FBV/N background responded poorly to HFD feedings than wild‐type mice, resulting in increases in adiposity/body weight and metabolic dysregulation (Cranford et al., 2016). Therefore, LGG pretreatment increased population of Bacteroidetes in the mouse gut microbiota, conferring less capacity of energy harvest and storage to the mice and thus less metabolic disturbance associated with concurrent down‐regulation of MCP1.

## 5 CONCLUSIONS

In conclusion, we have found that LGG as a dietary supplement decreased the F/B ratio of the gut microbiota and down‐regulated several pro‐inflammatory cyto‐kines/chemokines, but also increased the survival rate in response to experimental sepsis. Further studies, both in animal models or clinical trials, are warranted to address whether probiotic supplement can be adapted to clinical practice to reduce morbidity and mortality associated with sepsis.

## CONFLICT OF INTEREST

The authors declare that they have no conflict of interest.

## Data Availability

All data that support the findings of this study are available from the corresponding author on request.
